# Evaluation of the Efficacy and Safety of Canaloplasty and iStent Bypass Implantation in Patients with Open-Angle Glaucoma: A Review of the Literature

**DOI:** 10.3390/jcm10214881

**Published:** 2021-10-23

**Authors:** Kinga Gołaszewska, Joanna Konopińska, Iwona Obuchowska

**Affiliations:** Department of Ophthalmology, Medical University of Białystok, 15-089 Białystok, Poland; kin.golaszewska@gmail.com (K.G.); iwonaobu@wp.pl (I.O.)

**Keywords:** primary open angle glaucoma, minimally invasive glaucoma surgery, iStent, phacoemulsification with iStent, canaloplasty, phacocanaloplasty, intraocular pressure

## Abstract

The aim of the paper was to evaluate the efficacy and safety of minimally invasive antiglaucoma procedures: Canaloplasty and iStent bypass implantation with and without phacoemulsification in patients with primary open-angle glaucoma (POAG). A systematic review of the recent literature was performed based on the PubMed, Google Scholar, Web of Science and Scopus databases. The effectiveness of the procedures was evaluated based on the reduction of intraocular pressure (IOP) and the amount of antiglaucoma medication used before and after surgery. Safety of the treatments was evaluated based on the number of incidences of certain intraoperative and postoperative complications. Independent prospective randomized controlled trials (PRCTs) have demonstrated that both procedures, canaloplasty and iStent implantation, are effective in reducing IOP and the amount of antiglaucoma medication. Considering the safety profile of these procedures, both canaloplasty and iStent implantation are associated with low rates of postoperative complications and have similar safety profiles. Further studies are needed to confirm the results of our analysis, including a high-quality randomized controlled trial comparing canaloplasty and iStent bypass implantation

## 1. Introduction

Glaucoma, right after cataract, is the second most common cause of blindness in humans [1]. It affects more than 66 million people worldwide, and at least 6.8 million lesions are binocular. The primary goal of glaucoma therapy is to maintain useful visual function for the rest of the patient’s life [2]. The only modifiable risk factor for glaucoma development is elevated IOP [3]. Glaucoma surgery is effective in lowering IOP and maintaining a constant IOP level around the clock, but because of the risk of complications, it has been reserved for cases of moderate to severe glaucoma that do not respond to drug or laser therapy so far. The introduction of noninvasive or minimally invasive antiglaucoma procedures compared to classic trabeculectomy, has changed the thinking about glaucoma surgery which became increasingly common in cases of early and intermediate glaucoma. A decade ago, Saheb and Ahmed defined a new group of procedures, collectively referred to as minimally invasive glaucoma surgery (MIGS) [4]. They are characterized by five features: [1] Ab interno access through a corneal incision with conjunctival and scleral sparing; [2] low invasiveness of the procedure; [3] efficacy in lowering IOP; [4] an impact profile for patient comfort, and [5] rapid postoperative recovery. In 2014, the American Glaucoma Society and the U.S. Food and Drug Administration (FDA) also expanded this definition to include ab interno access procedures associated with very little impact on the conjunctiva or sclera or with omission of scleral preparation [5].

One of the methods used in MIGS procedures is implant placement. These include the iStent microstent from Glaukos (Glaukos Corporation, Laguna Hills, CA, USA). The iStent is made of heparin-coated titanium. It is implanted directly into the Schlemm’s canal, at the site of the highest concentration of collector channels (nasolabial quadrants), using a special feeder from an ab interno access without disturbing the conjunctival and scleral surfaces. The mechanism of the IOP-lowering effect of the iStent is to allow outflow of the aqueous fluid from the anterior chamber into the Schlemm’s canal, bypassing the site of highest outflow resistance, that is the trabecular meshwork.

Another of the antiglaucoma surgeries with a similar mechanism of action is canaloplasty. Lewis et al. [6] proposed a procedure involving the insertion of a catheter into Schlemm’s canal and then a suture to tighten its walls, which will result in improved outflow of aqueous fluid through the canal. Although traditional canaloplasty is performed ab externo and is not classified as MIGS, its variants, namely ab-interno canaloplasty (ABIC) [7] and mini-canaloplasty [8] are considered to be a minimally invasive surgeries. The common feature of this group of procedures is lowering IOP by improving the physiological mechanisms of water outflow. Canaloplasty begins with the insertion of viscoelastic into Schlemm’s canal, followed by passage of a microcephalus through the canal at a circumferential 360 degrees and placement of a circular suture to tighten the canal walls. The effect of this action is to widen the lumen of Schlemm’s canal and increase the tension of its walls. This counteracts the three main mechanisms responsible for the increase in resistance to outflow of the aqueous humor from the anterior chamber, namely the increase in resistance at the level of the pathologically altered beading [9], the collapse of the Schlemm’s canal lumen [10] and the collapse of the collector channels [11].

Our comparative analysis focuses on evaluating the efficacy and safety of canaloplasty and iStent bypass implantation in PAOG patients, based on a review of the recent literature. Both antiglaucoma surgeries have a similar mechanism of action, namely improving the outflow of aqueous humor through Schlemm’s canal, but differ in surgical technique. Implantation of the iStent seton is performed from an ab interno access without disturbing the conjunctiva and sclera, whereas canaloplasty is performed ab externo.

To date, many articles have been published comparing classic antiglaucoma procedures with MIGS procedures. There is an increasing trend in the emergence of studies comparing the efficacy and safety of MIGS with each other. To our knowledge, based on a thorough review of the literature, this is the first study to compare canaloplasty and iStent bypass implantation performed alone or simultaneously with phacoemulsification cataract surgery.

## 2. Materials and Methods

This systematic review was conducted and reported based on preferred reporting items for systematic reviews and meta-analyses (PRISMA) Statement, and the PRISMA network meta-analysis extension statement [1].

### 2.1. Search Strategy

A systematic review of the recent literature was performed based on the PubMed, Google Scholar, Web of Science and Scopus databases. We used the following key terms and phrases: “glaucoma”, “open angle glaucoma”, “primary open angle glaucoma”, “MIGS”, “iStent”, “trabecular micro-bypass stent”, “trabecular micro-bypass”, “canaloplasty”, “phacocanaloplasty”, “Schlemm’s canal surgery”. Summaries of articles were evaluated for information consistent with the topic of our analysis. Publications that were only available as abstracts or conference posters were excluded. After reading the abstract, full-text articles were selected related to the topic. No relevant articles were excluded based on text language and publication date. In addition, full-text translations were performed when necessary. Furthermore, was analyzed the literature included in the selected articles.

Studies with the following inclusion and exclusion criteria were considered for analysis.

### 2.2. Inclusion Criteria

- The study is a PRCT.
- The study involves patients with a diagnosis of POAG.
- One of the following surgical procedures was used: iStent bypass implantation with or without phacoemulsification, or canaloplasty with or without phacoemulsification.
- The study analyzes variables such as best corrected visual acuity (BCVA), IOP, and number of glaucoma drops used before and after surgery.
- The study shall include an adequate follow-up period.

### 2.3. Exlusion Criteria

- A case report, review, or experimental study.
- Studies describing partial results.
- Review-type papers.
- Studies not including all analyzed factors.

### 2.4. Risk of Bias Assessment

The methodological quality of included studies was evaluated independently by two authors (I.O., J.K.).

### 2.5. Data Extraction

The studies’ demographic details, participant characteristics, interventions, outcomes, and limitations were independently extracted by two authors (I.O., J.K.). If disagreements occurred, these were discussed and resolved through discussion.

**Figure 1:**
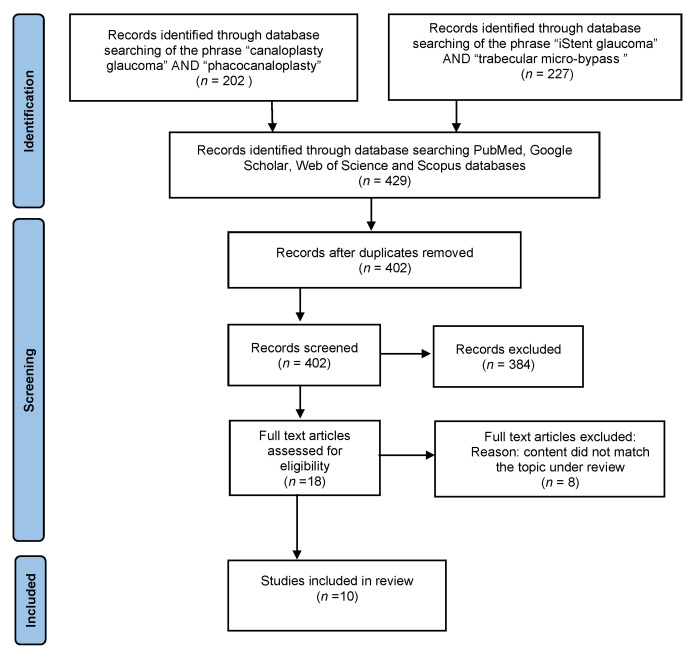
PRISMA flowchart of study selection process.

The PRISMA flowchart of literature selection in this systematic review is illustrated in Figure 1 [1]. We searched 429 articles containing studies on canaloplasty or iStent implant surgery. After removal of repeated studies, review articles, medical experiments, and case reports, 18 articles remained for full text-review. Eight papers that did not meet the inclusion criteria were also discarded. Finally, 5 RCTs on canaloplasty and 5 RCTs describing the safety and efficacy of iStent implantation were included in the systematic review (Table 1). The remaining publications found, regarding POAG and MIGS procedures, were used to outline the characteristics of our study and to introduce the topic of POAG.

## 3. Canaloplasty

Grieshaber et al. [12] conducted a prospective study involving a group of black Africans with advanced POAG. One eye in each patient was randomly selected for the study. Daily IOP curves were performed in all subjects on the day before surgery, which included IOP measurements at 8 am, 12 am, 4 pm, and 8 pm. The mean baseline IOP was very high at 45.0 +/− 12.1 mmHg.

Canaloplasty resulted in a sustained long-term reduction in IOP of 28.9 mmHg or 65.8% on average. The substantial reduction in IOP occurred in the early postoperative period. One week after surgery the mean IOP in all 60 eyes was 15.2 mmHg. These values remained stable during the three-year follow-up period of the study. Moreover, there was an additional decrease in mean IOP of 3 mmHg in 49 eyes approximately two years after surgery. Operative success at 36 months after canaloplasty as defined by three IOP levels—≤21, ≤18, and ≤16 mmHg, was—81%, 67.8%, and47.2%, respectively. Preoperative IOP, age, and gender had no effect on postoperative IOP.

The most common intra- or postoperative complication was transient microhyphema. Two (3.3%) patients developed Descemet’s membrane detachment, which adhered after two weeks. In the same number of patients, the microcele passed into the anterior chamber during cannulation, and in another two patients the microcele passed into the supravascular space. Only one patient had elevated IOP above 30 mmHg in the postoperative period [7].

In another study, Greishaber et al. [13] compared the size of the thread placed in Schlemm’s canal during canaloplasty. Group 1 consisted of patients with Prolene 6-0 suture, while group 2 consisted of patients with Prolene 10-0 suture. A 30% reduction in IOP without medication was achieved in 96.8% of group 1 and 97.8% of group 2, while a 50% IOP reduction was achieved in 55.6% of group 1 and 83.9% of group 2, respectively, at the end of follow up period. The most common postoperative complication observed in this study was microhyphema.

On the other hand, a prospective, multicenter study by Bull et al. [14], which compared canaloplasty (study group) to a combined operation of canaloplasty and phacoemulsification cataract removal (comparison group), showed somewhat less spectacular but equally satisfactory results in terms of IOP reduction. In eyes undergoing canaloplasty alone, a reduction in IOP values to 15.1 ± 3.1 mmHg was observed three years after surgery. Operative success at 36 months after canaloplasty as defined by the three IOP levels—≤21, ≤18, and ≤15 mmHg, were, respectively—40.5%, 36.5%, and 21.6%. In eyes qualified for the combined procedure, IOP decreased to 13.8 ± 3.2 mmHg three years after surgery.

The most common early postoperative complications were microhyphema (<1 mm anterior chamber blood level) and hyphema (>1 mm anterior chamber blood level). Elevated IOP and Descemet’s membrane detachment were reported slightly less frequently. In contrast, no case of hypotonia or shallowing of the anterior chamber was reported. In the group of late postoperative complications, cataract and transient IOP elevation were mainly observed.

The clinical study by Matlach et al. [15] focused on comparing the traditional procedure, trabeculectomy, with canaloplasty. Again, this study demonstrated the beneficial effect of canaloplasty on IOP reduction. At two years after canaloplasty, target IOP values of ≤20 mmHg or a 20% reduction in IOP were achieved in 39.1% of subjects. The mean IOP reduction was 9.3 ± 5.7 mmHg in these patients. Postoperative follow-up showed that canaloplasty is a safer procedure with a lower incidence of postoperative complications regarding hypotony, choroidal detachment, and IOP increase than trabeculectomy [15].

A recent clinical study by Rękas et al. [16] compared the efficacy results of phaco-canaloplasty with phaco-non-penetrating deep sclerectomy. A reduction in IOP was observed in both the study and comparison groups. With that said, a greater reduction in IOP was seen in the group after the phaco-canaloplasty procedure. In the study group, it was a 34% decrease from baseline. Whereas in the comparison group, the decrease was 25%. The most common complication in the study group was hyphema (58%). Patients who underwent phaco nonpenetrating deep sclerectomy were more likely to require additional procedures such as subconjunctival injection of 5-Fluorouracil, needling of the filter pad, and laser suture cutting [22].

All of the canaloplasty studies [12,13,14,15,16] reported a reduction in the mean number of antiglaucoma drops taken before the patient after surgery, compared with number of medication before surgery. BCVA assessment showed stabilization or improvement after canaloplasty in these patients.

Data on the effect of canaloplasty on BCVA, IOP, and antiglaucoma medication intake are summarized in Table 2.

## 4. iStent

In the study by Fea et al. [17], a reduction in IOP, to a mean value of 14.8 ± 1.2 mmHg, was observed 15 months after surgery, a 17.3% decrease from baseline IOP. As many as 67% of patients remained without pharmacological treatment in the period after iStent implantation. Two patients experienced iStent displacement, and no other postoperative complications were observed. Phacoemulsification with stent implantation was more effective in controlling IOP than phacoemulsification alone and the safety profiles were similar.

A study by Samuelson et al. [18] involving patients with early or intermediate POAG with IOP ≤ 24 mmHg on one to three medications compared a combined procedure consisting of iStent implantation and cataract extraction (study group) and a solo phacoemulsification (control group). A ≥20% IOP reduction was observed in 66% of patients and 72% of patients had postoperative IOP below 21 mmHg without medications 12 months after the combined procedure. Postoperative complications included, most commonly, stent obstruction, posterior pouch opacification, stent malposition, subconjunctival hemorrhage, epiretinal membrane, elevated IOP, and iris atrophy. Visual disturbances, iritis, dry eye syndrome, elevated IOP requiring treatment, and macular edema accounted for only 1% of complications [18].

A large, multicenter study by Craven et al. [19], involving patients with mild-to-moderate POAG with an IOP averaging 18.6 mmHg, that compared combined phaco + iStent surgery with solo phacoemulsification, showed similar results to the studies mentioned above. At 24 months after surgery, an IOP < 21 mmHg without medication was achieved in 71 (61%) patients in the study group, and a 20% reduction in IOP was achieved in 61 (53%) patients. The most common postoperative complications included posterior pouch opacification, elevated IOP, and stent obstruction. Anterior uveitis, conjunctival irritation caused by hypotensive medications were observed least frequently [19].

A study by Ahmed et. al. [20] compared the efficacy of Hydrus Microstent (Ivantis, Inc, Irvine, CA, USA) with 2 iStent Trabecular Micro Bypass devices. Satisfactory results were demonstrated including a reduction in IOP to 19.2 ± 2.4 mmHg without hypotensive medication. They also found that after 12 months of postoperative follow-up, 24.0% of patients with the 2-iStent did not require anti-glaucoma medications and 9.3% of patients had an IOP below 18 mmHg without medication [20]. The iStent implantation procedure showed a high safety profile in terms of postoperative complications. The most frequently observed was posterior pouch opacification of pseudophakia patients. In contrast, iStent displacement was not observed in any case.

Further confirmation of the effectiveness of the MIGS procedure in reducing IOP and hypotensive medications is the randomized study by Kozera et al. [21] A distinguishing feature of this study is the division of the study group (iStent implantation + phacoemulsification) and the control group (phacoemulsification solo) into two subgroups according to baseline IOP: the <26 mmHg group and the ≥26 mmHg group. The decrease in IOP after 24 months was greater in the study group, and the amount of antiglaucoma medication was significantly reduced compared with the control group. This study also confirmed the lower efficacy of the iStent’s hypotensive effect in patients with IOP ≥ 26 mmHg. This is attributed to collapse of Schlemm’s canal and decreased patency of the aqueous outflow tract from the anterior chamber of the eye. There were no significant differences in the safety profile between the study and control groups or between subgroups [21].

All of the iStent bypass implantation studies [17,18,19,20,21] reported a reduction in the mean number of antiglaucoma drops taken before the patient after surgery, compared with number of medication before surgery. BCVA assessment showed stabilization or improvement after surgery in these patients.

Data on the effect of iStent implantation on BCVA, IOP, and antiglaucoma medication intake are summarized in Table 3.

Table 4 summarizes data on complications occurring in the study groups in both the canaloplasty and iStent implantation papers.

## 5. Discussion

Surgical treatment of IOP should be considered when disease progression is observed despite conservative treatment. Despite their high efficacy in reducing IOP, filtering surgeries such as non-penetrating deep sclerectomy or trabeculectomy are often associated with complications related to filter bleb formation. This is the reason behind development of “blebless” minimally invasive procedures, or MIGS [23]. Because of its high safety, MIGS surgery has become an alternative to conservative treatment for many glaucoma physicians to treat patients with early glaucoma [24].

Our analysis showed that canaloplasty and iStent implantation are safe antiglaucoma procedures with low rates of both intraoperative and postoperative complications. The efficacy in lowering IOP in patients with mild to intermediate open-angle glaucoma is sufficiently high and long-lasting that it is possible to significantly reduce the antiglaucoma drops used after surgery.

To our knowledge, based on a thorough review of publications, this is the first analysis comparing a minimally invasive procedure like canaloplasty with a procedure from the MIGS group, iStent implantation. Based on the studies published so far, we can conclude that both procedures, canaloplasty and iStent implantation are effective in reducing IOP and in reduction in the dose of hypotensive medication. Both procedures have comparable effects on postoperative BCVA, preserving that of preoperative BCVA or even improving it. Considering the patient safety of these procedures, both canaloplasty and iStent are associated with few complications, which mostly resolve spontaneously without medical intervention.

Minimally and microinvasive surgery is dedicated to patients with early or intermediate POAG. The efficacy in IOP reduction is proportional with baseline parameters such as age, anterior chamber depth, as well as preoperative IOP. Accordingly, patients with IOP ≥ 26 mmHg achieved greater IOP reductions at follow-up than patients with IOP < 26 mmHg [21].

The study by Ferguson et al. [25] also confirmed the above thesis. The higher the baseline IOP was, the greater decrease in IOP was observed at the end of the follow-up period. Accordingly, in the group of patients with IOP ≥ 26 mmHg, a decrease in IOP of up to 11.3 mmHg was noted, compared to the IOP 22–25 mmHg group with a decrease of 7.7 mmHg and the IOP 18–21 mmHg group where a decrease of only 3.5 mmHg was noted [25]. However, on the other hand, patients with higher IOP also required more antiglaucoma medications before surgery than patients with lower IOP. Consequently, the reduction in medications used was greater and more satisfactory in patients with IOP < 26 mmHg. However the results of our analysis are no less consistent with what is commonly believed about so-called canal surgery and MIGS, which should not be expected to lower IOP as much as the antiglaucomatous classical surgery used in advanced glaucoma, with the creation of a full thickness filter flap [26].

Furthermore, both minimally invasive surgery and MIGS have a significantly higher safety profile than, for example, trabeculectomy, even though ab externo access is also used in canaloplasty. Trabeculectomy is the gold standard of antiglaucoma surgery, but it is associated with a high number of postoperative complications and requires more frequent postoperative intervention. With canaloplasty, the physiological drainage of the aqueous humor through the bead canal is increased by opening or widening of the Schlemm’s canal, without the need to create a functional filtering bleb [27]. Therefore, the risk of potential pathway for pathogens to enter the eyeball and cause inflammation inside the eyeball is eliminated as well.

When considering intraoperative and postoperative safety, it is worth noting that iStent implantation reduces the number of procedures from two to one and therefore also reduces the risk of intraoperative and postoperative complications.

In addition, the iStent placed in the isthmus provides an additional pathway for the outflow of the aqueous humor from the anterior chamber of the eye leading to lower IOP and slowing the progression of glaucomatous neuropathy.

Although our analysis can be very helpful in qualifying a patient for a particular treatment option, it also contains some limitations. First, we included only PRCTs of high quality, but the number of these studies is still relatively low. Second, there is a large discrepancy in the number of eyes involved in the studies, ranging from 24 to 240, making the groups compared inhomogeneous. In addition, no clinical work comparing the efficacy and safety of canaloplasty and iStent implantation between each other in a single study has been published to date. Therefore, the conclusions of our analysis are somewhat “indirect” and are derived from comparing papers describing the results of one method to papers describing the results of the other method. Thus, what is needed is a well-designed randomized clinical trial comparing both procedures: Canaloplasty and bypass iStent implantation in terms of efficacy and safety for the patient.

## 6. Conclusions

Based on existing studies, our analysis showed that both procedures, canaloplasty and iStent implantation, are effective in reducing IOP and the amount of antiglaucoma medications. Considering patient safety, both canaloplasty and iStent implantation have low complication rates and similar safety profiles. Because of the minimally invasive nature of iStent implantation, this procedure could be considered as a routine treatment in glaucoma patients who qualify for elective cataract surgery. Despite some limitations, our analysis may help in deciding the patient’s eligibility for a specific type of surgical treatment. Further studies including a high-quality randomized controlled trial comparing canaloplasty and iStent bypass implantation are needed to confirm the results of our analysis.

## Figures and Tables

**Table 1:** Characteristics of the prospective randomized control trials included in the analysis.

Author	Year	Setting	Type of Surgery of Studied Group	Sample Size: Studied Group (Control Group)	Follow-Up (Months)
Grieshaber et al. [12]	2010	Southern Africa	Canaloplasty	60 *	30.6 ± 8.4
Grieshaber et al. [13]	2010	Southern Africa	Canaloplasty	90 **	15
Bull et al. [14]	2011	Multicenter	Canaloplasty	109 **	36
Matlach et al. [15]	2015	Germany	Canaloplasty	30 (32)	24
Rękas et al. [16]	2015	Poland	Canaloplasty + Phacoemulsification	29 (30)	12
Fea et al. [17]	2010	Italy	iStent + Phacoemulsification	12 (24)	15
Samuelson et al. [18]	2010	Multicenter	iStent + Phacoemulsification	117 (123)	24
Craven et al. [19]	2012	Multicenter	iStent + Phacoemulsification	117 (123)	24
Ahmed et al. [20]	2019	Multicenter	2 by-pass iStent	77 (75)	12
Kozera et al. [21]	2021	Poland	iStent + Phacoemulsification	44 (36)	24

* Only studied group; ** Both groups included, no separate data.

**Table 2:** Mean pre- and postoperative values of best corrected visual acuity, intraocular pressure, and number of antiglaucoma medications used before and after canaloplasty.

Author	Mean Preoperative BCVA *	Mean Preoperative IOP **	Preoperative Number of Applied Eye Drops	Mean BCVA * after Follow-Up Period	Mean IOP ** after Follow-Up Period	Number of Applied Eye Drops after Follow-Up Period
Grieshaber et al. [12]	0.61 ± 0.42	45.0 ± 12.1 mmHg	Without eye drops	0.58 ± 0.31	13.3 ± 1.7 mmHg	Without eye drops
Grieshaber et al. [13]	LOD ****	42.7 ± 12.5 (Prolene 6-0) 45.0 ± 12.1 (Prolene 10-0)	Without eye drops	LOD ****	19.2 ± 6.4 mmHg (Prolene 6-0) 16.4 ± 4.9 (Prolene 10-0)	Without eye drops
Bull et al. [14]	0.22 ± 0.25	23.0 ± 4.3 mmHg	1.9 ± 0.7	0.20± 0.26	15.1 ± 3.1 mmHg	0.9 ± 0.9
Matlach et al. [15]	0.08–0.40 logMAR ***	23.7 ± 5.1 mmHg	2.6 ± 1.6	0.20 ± 0.26 logMAR	14.4 ± 4.2 mmHg	0.9 ± 1.1
Rękas et al. [16]	0.74 ± 0.70 logMAR ***	19.0 ± 6.9 mmHg	2.64 ± 0.68	0.11 ± 0.17 logMAR	12.6 ± 2.7 mmHg	0.27 ± 0.67

* BCVA—best corrected visual acuity; ** IOP—intraocular pressure; *** logMAR—log of the minimum angle of resolution.; **** LOD—lack of data.

**Table 3:** Mean pre- and postoperative values of best corrected visual acuity, intraocular pressure, and number of antiglaucoma medications used before and after iStent implantation.

Author	Mean Preoperative BCVA *	Mean Preoperative IOP **	Preoperative Number of Applied Eye Drops	Mean BCVA * after Follow-Up Period	Mean IOP ** after Follow-Up Period	Number of Applied Eye Drops after Follow-Up Period
Fea et al. [17]	no better than 0.6	17.9 ± 2.6 mmHg	1.9 ± 0.7	LOD ***	14.8 ± 1.2 mmHg	0.4 ± 0.7
Samuelson et al. [18]	no better than 0.5	18.4 ± 3.2 mmHg	1.5 ± 0.6	0.36 ± 0.23 log MAR	mean reduction in IOP compared with the preoperative unmedicated baseline IOP was 8.4 ± 3.6 mmHg	0.2 ± 0.6
Craven et al. [19]	0.75 ± 0.25	18.6 ± 3.4 mmHg	1.6 ± 0,8	0.75 ± 0.25	17.1 ± 2.9 mmHg	0.3 ± 0.6
Ahmed et al. [20]	0.83	19.1 ± 3.6 mmHg	2.7 ± 0.8	BCVA loss > 2 lines at 12 months, *n* (%)1 (1.3)	19.2 ± 2.4 mmHg	Without eye drops
Kozera et al. [21]	0.56 ± 0.23	22.04 ± 1.64 mmHg	1.32 ± 0.55	0.95 ± 0.12	15.57 ± 2.13 mmHg	0.32 ± 0.55

* BCVA—best corrected visual acuity; ** IOP—intraocular pressure; *** LOD—lack of data.

**Table 4:** Main postoperative complications after canaloplasty and iStent implantation.

	Author	Grieshaber et al. [12] *n* (%)	Grieshaber et al. [13]	Bull et al. [14] *n* (%)	Matlach et al. [15] *n* (%)	Rękas et al. [16] *n* (%)	Fea et al. [17] *n* (%)	Samuelson et al. [18] *n* (%)	Craven et al. [19] *n* (%)	Ahmed et al. [20] *n* (%)	Kozera et al. [21] *n* (%)
Complication											
Stent obstruction		NA	NA	NA	NA	NA	NR	4 (4%)	5 (4.3%)	NR	NR
Stent malposition		NA	NA	NA	NA	NA	2	3 (3%)	3 (2.6%)	0	NR
Elevated IOP		1 (1.67%)	4 (4.4%)	6 (5.5%)	1 (3.4%)	NR	NR	2 (2%)	5 (4.3%)	NR	NR
Posterior capsule opacification		NR	NR	NR	NR	NR	NR	3 (3%)	7 (6%)	5%	4 (9.1%)
Blurry vision or visual disturbance		NR	NR	NR	NR	NR	NR	1 (1%)	4 (3.4%)	1	NR
Microhyphema		42 (70%)	25 (27.8%)	14 (12.8%)	NR	10 (34.5%)	NR	NR	NR	NR	5 (11.4%)
Hyphema		7 (22.3%)	NR	6 (5.5%)	NR	17 (58.0%)	NR	NR	NR	NR	NR
Descemet’s membrane detachment		2 (3.33%)	1 (1.1%)	4 (3.7%)	NR	1 (3.4%)	NR	NR	NR	NR	NR
Cataract		NR	NR	17 (19.2%)	NR	NR	NR	NR	NR	1	NR
Iritis		NR	NR	NR	1 (3.4%)	2 (6.9%)	NR	1 (1%)	1 (0.9%)	NR	1 (2.3%)
Hypotony		NR	NR	NR	NR	NR	NR	NR	NR	NR	NR

IOP—intraocular pressure; NA—not applicable; NR—not reported.

## Data Availability

All the materials and information will be available upon an e-mail request to the corresponding author.
